# CD45RA Distinguishes CD4+CD25+CD127−/low TSDR Demethylated Regulatory T Cell Subpopulations With Differential Stability and Susceptibility to Tacrolimus-Mediated Inhibition of Suppression

**DOI:** 10.1097/TP.0000000000001278

**Published:** 2016-07-28

**Authors:** Rebeca Arroyo Hornero, Gareth J. Betts, Birgit Sawitzki, Katrin Vogt, Paul N. Harden, Kathryn J. Wood

**Affiliations:** ^1^ Nuffield Department of Surgical Sciences, Oxford University, John Radcliffe Hospital, Oxford, United Kingdom.; ^2^ Charité-Universitätsmedizin Berlin, Campus Virchow, Institut f. Medizinische Immunologie, Berlin, Germany.; ^3^ Oxford Transplant Centre, Oxford University Hospitals NHS Trust, Oxford, United Kingdom.

## Abstract

**Background:**

Adoptive transfer of forkhead box protein (FOX)3^+^ regulatory T (Treg) cells offers a promising strategy to reduce damage to an allograft by the recipient's immune system. Identification of cell surface markers sufficient to purify Treg cells expanded ex vivo to remove cellular contaminants requires optimization. Furthermore, the expanded Treg must be able to survive, expand, and suppress in allograft recipients exposed to immunosuppressants, such as tacrolimus (TAC). Reduced CD127 expression enhances identification of Treg in the human CD4^+^CD25^+^ population. CD45RA expression identifies naive CD4^+^CD25^+^ Treg with an enhanced stability of Treg phenotype.

**Methods:**

We combine an analysis of CD45RA, CD25, and CD127 expression to identify subpopulations of CD4^+^CD127^−/lo^CD25^+^ cells. Regulatory T cells were sorted according to expression of CD25 and CD45RA and expanded in the presence of a physiological relevant concentration of TAC. Regulatory T cell–specific demethylation region (TSDR) demethylation, FOXP3 expression, and suppression were analyzed.

**Results:**

CD4^+^CD127^−/lo^CD25^+^CD45RA^+^ Treg cells had a stable TSDR demethylated FOXP3^+^ phenotype after expansion whereas CD4^+^CD127^lo/−^CD25^+^CD45RA^−^ Treg cell lost the TSDR demethylated phenotype. CD45RA^−^ Treg had a greater capacity to suppress after expansion with TAC.

**Conclusions:**

Although CD45RA^−^ Treg retained a greater suppressive capacity when expanded with TAC, the marked loss of the TSDR demethylated status highlights the potential for loss of stability of these cells in transplant recipients treated with TAC based immunosuppression. We show that a population of CD4^+^CD127^−/lo^CD45RA^+^ Regulatory T cell may offer the best compromise between susceptibility to loss of suppression when exposed to TAC and maintenance of a TSDR demethylated phenotype following in vitro expansion.

## Abstract 2

Following in vitro expansion with tacrolimus, CD4+CD127-/loCD25+CD45RA- Treg had a greater suppressive capacity vs the CD45RA+ counterpart Treg. Nevertheless, CD45RA+ Treg had a more stable TSDR demethylated FOXP3+ phenotype vs the CD45RA- Treg counterpart. CD4+CD127-/loCD25+CD45RA+ Treg offer the best compromise following expansion. Supplemental digital content is available in the text.

Drugs that are used to suppress antiallograft immunity in kidney transplant recipients (KTR) are nephrotoxic and increases the incidence of malignancy and cardiovascular pathology.^1^ Investigations are underway to use immune regulatory cellular therapy to induce immunological unresponsiveness to donor alloantigens,^2^ which may permit a reduction of immunosuppression in the longer term. Infusion of Treg cells is 1 proposed cellular therapy undergoing investigation in KTRs.^3^ Regulatory T cells infused into transplant patients must be able to survive, retain suppressive function, and potentially continue to expand to replace Treg cells that die as part of natural cell turnover in the presence of immunosuppressants, including calcineurin inhibitors (CNIs), such as tacrolimus (TAC). Regulatory T cells require low doses of IL-2 to expand and become functionally active.^4,5^ CNIs interfere with IL-2 signaling and have been implicated to reduce Treg numbers posttransplant.^6,7^ The ONE study clinical trial is examining Treg cell adoptive transfer therapy in combination with withdrawal of steroids and mycophenolate mofetil immunosuppression in KTRs; however, the withdrawal of TAC therapy is not currently planned and therefore the identification of Treg populations that remain suppressive in the presence of TAC may be critical to implementing Treg cell therapy successfully in an allograft transplant setting.

Low or absent CD127 cell surface expression in combination with CD25 expression is an important and long-established marker to aid the distinction of Treg from T effector (Teff) cells that express higher levels of CD127 in humans. Forkhead box protein (FOX)3 expression inversely correlates with CD127 expression and has been shown to directly regulate CD127 expression through binding the CD127 promoter.^8,9^ Human CD25^+^CD127^−/lo^ Treg cells have been demonstrated to be highly suppressive in vivo in a humanized mouse model.^10,11^ Live human CD25^+^CD127^−/lo^ Treg cells may be purified and subsequently validated as bona fide Treg by ensuring a high enrichment of FOXP3^+^ cells and demethylation of the Treg cell-specific demethylated region (TSDR) of the FOXP3 promoter.^12^ The latter finding is important because downregulation of CD127 and upregulation CD25 and FOXP3 expression by human activated conventional CD4^+^ T cells are possible.^12^ Distinct populations within total CD4^+^ T cells have also been identified using CD25 and CD45RA cell surface markers, each with differential FOXP3 expression and suppressive capacity.^13,14^ Here we demonstrate that CD4^+^CD25^+^CD127^−/lo^ Treg sorted according to differential CD45RA expression distinguishes Treg subpopulations that, after in vitro expansion with a physiological concentration of TAC, have a differential TSDR demethylated phenotype and suppressive function. These observations will inform the design of protocols to deliver Treg cellular therapy to transplant patients receiving CNIs, including TAC.

## MATERIALS AND METHODS

### Flow Cytometric Phenotyping

Cells were stained with 7-AAD viability dye, anti-CD3 eFluor450, anti-CD4 PE-eFluor 647 (eBioscience), anti-CD4 electron coupled dye (Beckmann Coulter), anti-CD4 fluorescein isothiocyanate (FITC), anti-CD3 PE, anti-CD3 APC-Cy7, anti-CD8 PE, anti-CD8 APC-Cy7, anti-CD25 PECy7, anti-CD127 PE, and anti-CD27 FITC (BD), anti-CD45RA FITC, FOXP3 Alexa Fluor 647 (BioLegend) specific antibodies. FOXP3 intracellular staining was performed using FOXP3 staining buffers (eBioscience). Data were acquired using a fluorescence activated cell sorting (FACS)CantoII and analysed using FACSDiva software (BD) and Flojo software (Treestar).

### Treg Sorting Strategies

Peripheral blood mononuclear cells (PBMC) were isolated from blood cones obtained from healthy donors (NHS Blood and Transplant [NHSBT] UK) by LSM 1077 (PAA) gradient centrifugation and then incubated with CD25^+^ Microbeads to derive CD25^+^ enriched cells using a LS column (Miltenyi Biotech). CD25^+^ cells were stained with monoclonal antibodies and Treg cell subsets were isolated by FACS using a BD FACSAria I cell sorter.

**FIGURE 1:**
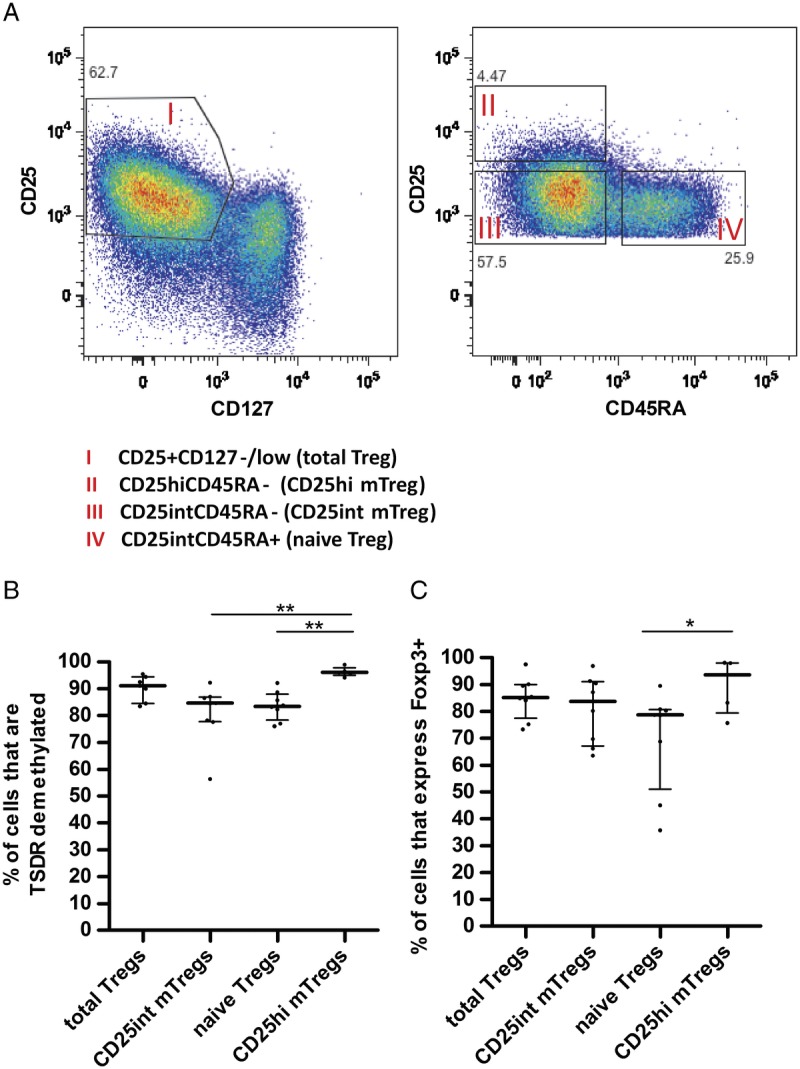
Treg phenotyping. A, Gating strategies to isolate total Treg (CD127^−/low^CD25^+^); naive Treg (CD127^−/low^CD25^int^CD45RA^+^); CD25^int^ mTreg (CD127^−/low^CD25^int^CD45RA^−^); and CD25^hi^ mTreg (CD127^−/low^CD25^hi^CD45RA^−^). B, Percentages of cells with demethylated TSDR (n = 11) and **(**C) percentage of cells that express FOXP3 (n = 12) before expansion (Mann-Whitney *U* test, *P < 0.05, **P < 0.01, ***P < 0.001). Median with interquartile range is represented.

The total CD4^+^CD127^−/low^CD25^+^ Treg cell (total Treg cells) population (purity: mean, 91.9% (82.8-95.9%) of total sorted live CD4^+^ cells) was sorted into 3 phenotypically distinct subpopulations, based on expression of CD25 and the naive T cell marker CD45RA: CD127^−/low^CD25^int^CD45RA^−^ (CD25^int^ memory Treg [mTreg]) and CD127^−/low^CD25^hi^CD45RA^−^ memory Treg (CD25^hi^ mTreg); and CD127^−/low^CD25^int^CD45RA^+^ naive Treg (naive Treg) (Figure 1A). Purity of sorted populations was mean 87.6% (81.0-95.3%) CD25^int^ mTreg, mean 73.8% (63.9-84.7%) CD25^hi^ mTreg and mean 75.6% (36.9-94.9%) naive Treg of total live CD4^+^ cells.

### In Vitro Expansion of Treg

Expansion protocol was adapted from previous protocols with minor modifications.^10^ Sorted cells were expanded in Roswell Park Memorial Institute 1640 media supplemented with l-glutamine, penicillin/streptomycin, sodium pyruvate, and 10% of human AB pooled serum, in the presence of recombinant human IL-2 (1000 U/mL) (Novartis) and Dynabead Human T-activator CD3/CD28 (Life Technologies) in a 1:1 cell to bead ratio over two 7-day rounds of expansion. During the second round of expansion, 8 ng/mL TAC (Sigma) dissolved in dimethyl sulfoxide (DMSO) (Sigma), based on the published trough level in patients with stable liver or kidney allografts,^6,15,16^ or empty DMSO control added to cell media (final concentration of DMSO in culture was 1.92 × 10^−4^% volume).

### In Vitro Suppression Test

Expanded Treg suppressive capacity was assessed in 72 hours in vitro assays in the absence of TAC. Variable numbers of Treg were incubated with 10 × 10^4^ autologous PBMC labelled with 1 μM VPD450 (BD) proliferation dye in triplicate wells containing 2 × 10^4^ αCD3/αCD28 beads in 96-well plates. Labeled PBMC cultured alone in the presence of αCD3/αCD28 beads were used as a negative control for suppression. After a 72-hour incubation, cocultures were stained to distinguish CD4^+^ and CD8^+^ cells, and violet proliferation dye dilution was analyzed by flow cytometry. The percentage of PBMC suppression was calculated by using division index of cocultures containing Treg and PBMC compared with division index of PBMC alone, according to the following formula (1 − (div.index Treg + PBMC/div.index PBMC)). Data were analyzed with FlowJo software version 9.5.3. The program calculates division index as the average of cell divisions that a cell in the original population has undergone.

### TSDR DNA Methylation Analysis

Regulatory T cell–specific demethylation region DNA methylation analysis was performed as previously described^17^ using genomic DNA isolated from freshly sorted or expanded Treg cells using the QIAamp DNA Mini Kit (QIAGEN, Hilden, Germany). A minimum of 60-ng bisulfite-treated (EpiTect; Qiagen) genomic DNA was used in a real-time polymerase chain reaction (PCR) to quantify the Foxp3 TSDR. Real-time PCR was performed in a final reaction volume of 20 μL containing 10-μL FastStart universal probe master (Roche Diagnostics, Mannheim, Germany), 50-ng/μL lamda DNA (New England Biolabs, Frankfurt, Germany), 5-pmol/μL methylation or nonmethylation-specific probe, 30-pmol/μL methylation or nonmethylation-specific primers, and 60-ng bisulfite-treated DNA or a respective amount of plasmid standard. The samples were analyzed in triplicates on an ABI 7500 cycler and reported as % T cells with demethylated TSDR region.

### Cytokine Analysis

Two-week expanded Treg cells were thawed and expanded further for 2 days in DMSO or TAC containing media with IL-2 (1000 U/mL) and with 2 × 10^4^ αCD3/αCD28 beads (1:5 bead:Treg) or without bead stimulation as a negative control. Supernatants were then centrifuged and stored at −80°C until analyzed. IL-10, IL-17A, and IFN-γ levels were determined using enzyme-linked immunosorbent assay according to manufacturers' protocol (BD for IL-10 or eBiosciences for IL-17A and IFN-γ).

### Figures and Statistical Analysis Software

Graphs were produced and statistical analyses performed using Graph Prism 5.00 (GraphPad Software, San Diego, CA) and IBM SPSS statistics v22 software.

## RESULTS

### Characterization of TSDR Demethylation Status and Foxp3 Expression in 4 Freshly Isolated Treg Subpopulations

Preliminary analysis demonstrated a large variation in phenotype, suppressive function, expansion, and susceptibility to TAC-induced alteration of Treg function between CD4^+^CD25^+^CD127^−/lo^ cells (total Treg cells) isolated from different healthy human donors (data not shown). It is possible that heterogeneity in total CD4^+^CD25^+^CD127^−/lo^ Treg cells between donors accounted for these variations. Further experiments were therefore performed by costaining with CD45RA in an attempt to define a more homogeneous subpopulation of Treg present consistently in different donors. Total Treg cells were sorted into naive Treg cell, CD25^int^ mTreg cells, and CD25^hi^ mTreg cells as described in the Methods (Figure 1A).

Approximately 91% of total Treg had a demethylated TSDR before expansion (median, 91.1%). A significantly larger proportion of the CD25^hi^ mTreg subpopulation showed a demethylated TSDR (median, 96.2%) compared with naive Treg (median, 83.5%) and CD25^int^ mTreg (median, 84.6%) (Figure 1B).

A high proportion of cells expressing FOXP3 protein corresponded to a high proportion of cells with a demethylated TSDR in sorted populations (Figures 1B and C., respectively). Similar to TSDR demethylation results, the proportion of cells expressing Foxp3 tended to be higher in CD25^hi^ mTreg (median, 93.6%) compared with CD25^int^ mTreg (median, 83.7%), naive Treg (median, 78.7%), and total Treg (median, 85.2%) (Figure 1C).

### TAC Reduces the In Vitro Expansion Capacity of All Treg Cell Subpopulations Equally

It is possible that successful induction of immunological unresponsiveness after transfusion of Treg cells into patients will require further expansion in vivo to both control allospecific immunity adequately and replace Treg cells that die as part of natural cell turnover. In this circumstance, Treg cells will need to expand in vivo despite exposure to pharmacological immunosuppression.

**FIGURE 2:**
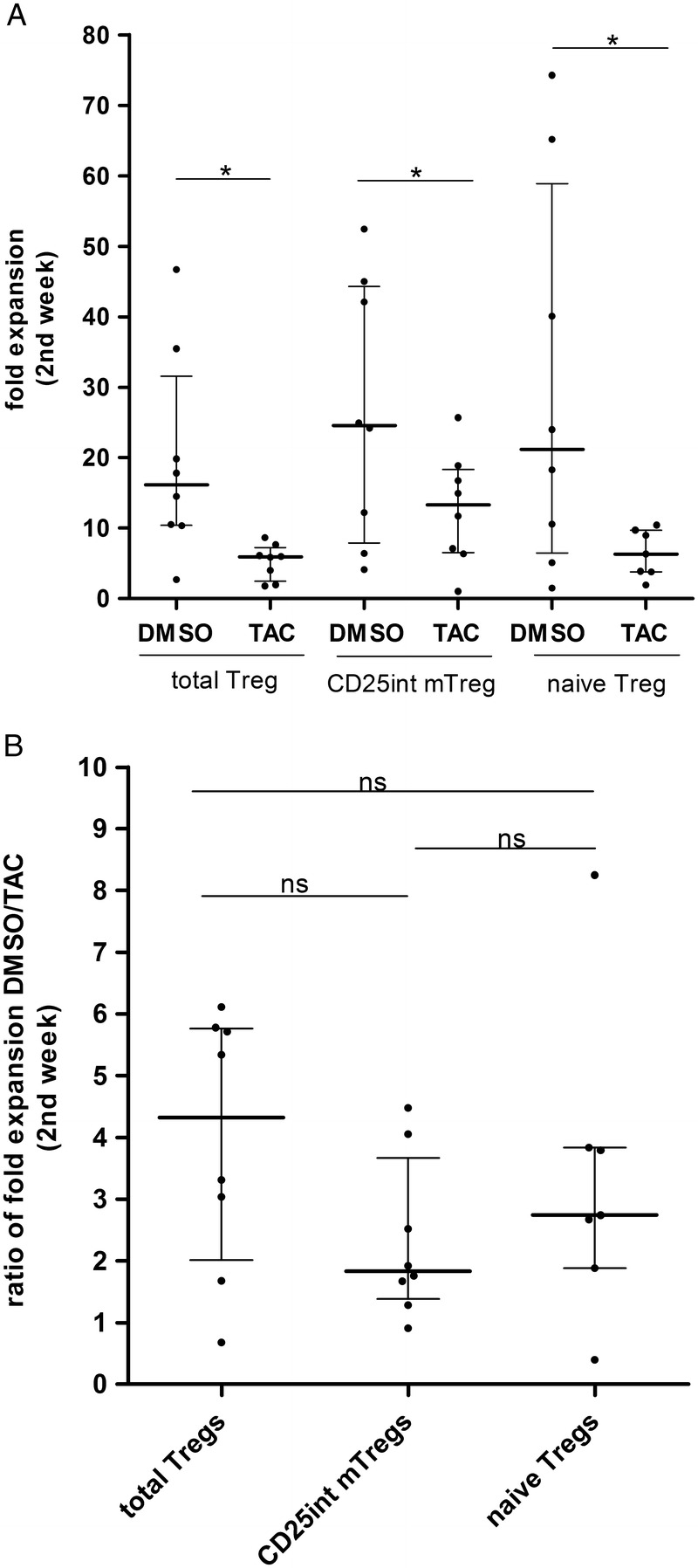
Expansion capacity of Treg cell populations. A, Fold expansion in each Treg cell population was measured after in vitro expansion in the presence of DMSO or TAC (Wilcoxon-matched pair test), comparing day 7 with day 14 cell numbers. B, Ratio of proliferation between each Treg cell population incubated with DMSO or TAC (Mann-Whitney *U* test). n = 12 donors are shown (*P < 0.05, **P < 0.01, ***P < 0.001). Median with interquartile range is represented.

To investigate whether TAC differently affected the expansion of Treg subpopulations, we analyzed the effect of TAC on cell expansion in the final 7 days of a 14-day culture, comparing cell numbers at day 7 with day 14, when cells were exposed to TAC (8 ng/mL) or DMSO vehicle control. CD25^hi^ mTreg were highly anergic and could not be expanded sufficiently to perform experiments. As expected, TAC significantly reduced expansion of all Treg populations. CD25^int^ mTreg expanded a median of 25- and 13-fold; naive Treg by a median of 21- and 6-fold; and total Treg cells expanded a median of 16- and 6-fold in the presence of DMSO or TAC, respectively (Figure 2A). Analysis of the ratio of proliferation between Treg incubated with DMSO and TAC demonstrated that TAC did not inhibit proliferation of any 1 Treg cell subpopulation more than any other (Figure 2B).

### Treg Phenotype in Different Subpopulations After Expansion Without TAC

Next, the stability of the phenotype of total Treg cells, naive Treg cells, and CD25^int^ mTreg cells after expansion without TAC was examined. FOXP3 expression and TSDR demethylation were measured on day 0 and day 14, suppressive function was determined on day 14.

**FIGURE 3:**
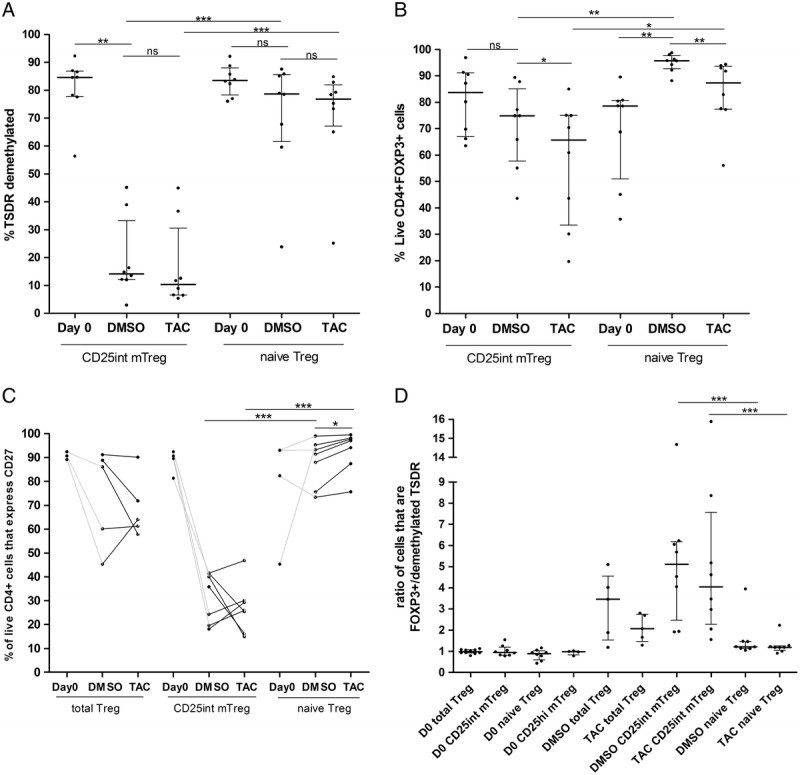
Foxp3 expression and demethylation status of the TSDR region of expanded Treg cell subsets. A, Percentages of TSDR demethylation (n = 8 donors); B, cells expressing Foxp3 (n = 8 donors); C, cells expressing CD27 (n = 7 donors); and D, ratio of cells that express FOXP3 and with a demethylated TSDR were measured in each Treg cell population before and after in vitro expansion in the presence of DMSO or TAC. For statistical analysis, Wilcoxon-matched pair test was performed comparing, CD27 expression, Foxp3 expression, TSDR demethylation or FOXP3^+^/demethylated TSDR ratio between the respective Treg population after being exposing to DMSO or TAC, and the Mann-Whitney *U* test was used to compare between cell populations (*P < 0.05, **P < 0.01, ***P < 0.001). Median with interquartile range is represented.

Before expansion, the majority of naive Treg and CD25^int^ mTreg expressed FOXP3 and possessed a demethylated TSDR (Figure 1). Naive Treg and CD25^int^ mTreg cells showed differential stability of this Treg cell phenotype during expansion in DMSO control media. A significantly higher proportion of CD25^int^ mTreg cells did not have a demethylated TSDR phenotype (median, 14.2% remaining demethylated) compared to the naive Treg cell subpopulation (median, 78.7% remaining demethylated) (Figure 3A, *P* < 0.05) by day 14. This finding correlated with a substantial reduction in the population that expressed CD27, a marker known to be associated with highly suppressive Treg,^18^ by CD25^int^ mTreg compared with naive Treg expanded in DMSO control media (Figure 3C).

CD25^int^ mTreg maintained similar FOXP3 protein expression before and after expansion (median, 83.7% and 74.9%, respectively) in DMSO control media, whereas the proportion of naive Treg that expressed FOXP3 increased (median, 78.7% to 95.8% after expansion) (Figure 3B). As the extensive loss of TSDR demethylation status in the CD25^int^ mTreg population is not reflected by reduced FOXP3 expression, it appears that the CD25^int^ expanded population contains a greater proportion of activated Teff cell or induced Treg (iTreg) cell that may transiently express FOXP3 without having a demethylated TSDR. A reduced level of TSDR demethylation alongside a sustained FOXP3 expression altered the ratio of FOXP3 expressing cells compared with TSDR demethylated cells after expansion in the CD25^int^ mTreg population (Figure 3D).

When comparing preexpanded and cells expanded in DMSO, total Treg cells showed a level of TSDR demethylation (median, 25.2% retain) and Foxp3 expression (median, 87.4% retain) that was intermediate to naive Treg and CD25^int^ mTreg subpopulations (data not shown) to manifest an enhanced ratio of FOXP3 expression to TSDR demethylation (Figure 3D), that is probably due to inclusion of CD25^int^ mTreg when sorted on day 0.

**FIGURE 4:**
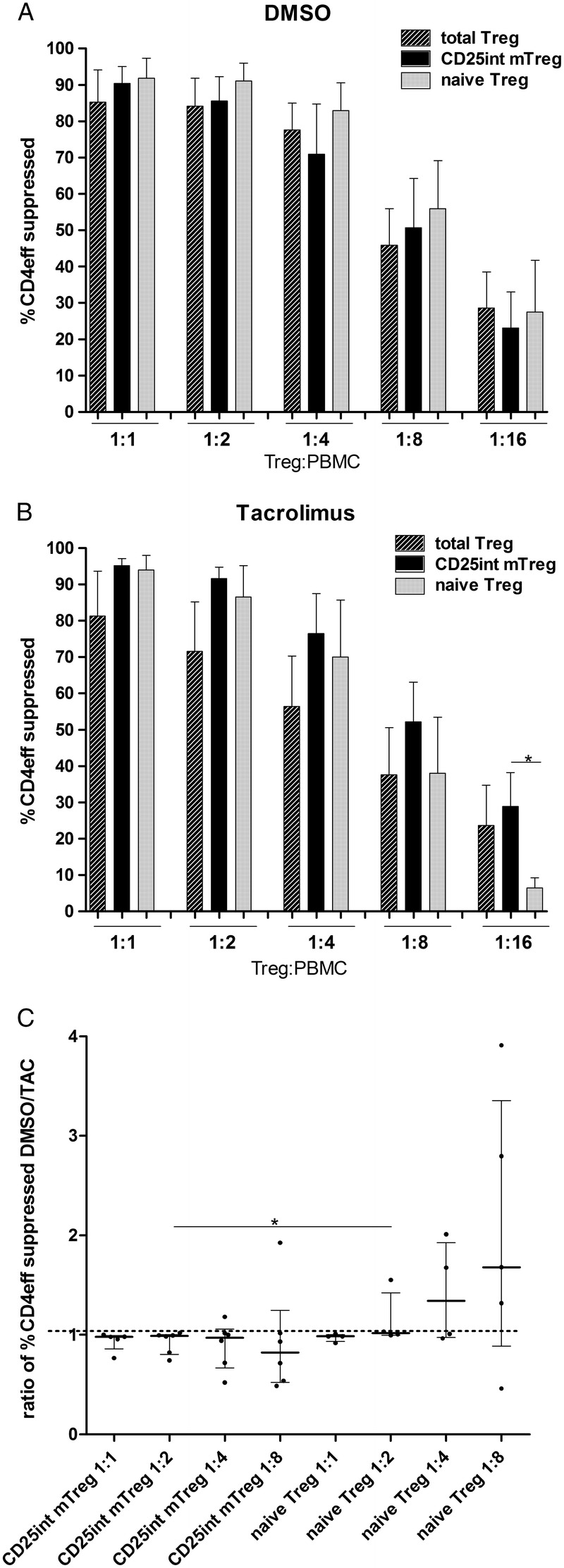
In vitro suppressive capacity of Treg cell populations. A, Suppressive capacity of CD25^int^ mTreg and naive Treg (n = 6 donors), and total Treg (n = 5 donors) after in vitro expansion in DMSO control media. B, Suppressive capacity of CD25^int^ mTreg and naive Treg (n = 6 and n = 5 donors, respectively), and total Treg cells (n = 5 donors) after in vitro expansion in the presence of TAC. Percent suppression of CD4^+^ effector cell proliferation based on division index of PBMCs compared with the proliferation of PBMCs cultured in the absence of suppressor cells. Each data point is the average of 3 replicate wells in the suppression assay of each donor. Mann-Whitney *U* test used to compare suppression between Treg subpopulations (*P < 0.05). Mean with SEM is represented. C, Ratio of percentage suppression between Treg expanded in DMSO and TAC containing media, using titrated numbers of Treg to Teff cells. (n = 6 donors; Mann-Whitney *U* test, *P < 0.05). Median with interquartile range is represented.

Despite presenting higher levels of TSDR demethylation and Foxp3 expression, expanded naive Treg cells were not significantly more suppressive than CD25^int^ mTreg cells (Figure 4A); suppression was similar between total Treg cells, CD25^int^ mTreg cells, and naive Tres cells after expansion in DMSO control media (Figure 4A). This indicates that those CD25^int^ mTreg cells that express FOXP3 but do not retain a demethylated TSDR are iTreg cells that are capable of suppressing.

### Effect of TAC

The effect of TAC on Treg phenotype and suppressive function was analyzed. The effect of TAC on IL-2 production is not examined here, as although TAC would be expected to suppress IL-2 production, exogenous IL-2 was added to cultures.

Although TAC did not alter the expression of CD27 in total Treg or CD25^int^ mTreg populations (Figure 3C) compared with DMSO control media, CD27 expression was slightly increased by naive Treg exposed to TAC (*P* < 0.05, Figure 3C).

Tacrolimus has been shown to downregulate FOXP3 expression, whereas TSDR demethylation remained stable in allograft recipient patients.^6,19^ In keeping with these findings, we observed that the percentage of CD25^int^ mTreg and naive Treg cells (Figure 3A) with demethylated TSDR was not different between cells expanded in TAC or DMSO control containing media. In contrast, cells expanded with TAC showed reduced FOXP3 expression in naive Treg and CD25^int^ mTreg cell populations (*P* < 0.01, *P* < 0.05, respectively; Figure 3B).

Total Treg, CD25^int^ mTreg, and naive Treg showed equal suppressive function after expansion in DMSO (Figure 4A); however, suppressive function of naive Treg expanded in TAC appeared to be partially abrogated compared with CD25^int^ mTreg, when lower ratios of Treg-Teff were examined (Figure 4B; 1:16 ratio *P* < 0.05). When analyzing the suppressive capacity of Treg generated from 6 donors independently, a clear trend was observed that TAC reduced suppression of the naive Treg subpopulation in a larger proportion of donors compared with the CD25^int^ mTreg cell subpopulation (2-sided Fisher exact test: *P* = 0.08). Tacrolimus impaired suppressive capacity of CD25^int^ mTreg in only 1 of 6 donors (**Figure 1A, SDC,** http://links.lww.com/TP/B286); in contrast, in 4 of 5 donors, naive Treg showed reduced suppressive activity after expansion with TAC (sufficient numbers of naive Treg expanded with TAC could not be obtained from 1/6 donors) (**Figure 1B, SDC,** http://links.lww.com/TP/B286). An examination of the ratio of suppression by Treg expanded in DMSO and TAC showed that the naive Treg population was more susceptible to TAC mediated reduction of suppression than the CD25^int^ mTreg population (Figure 4C).

### CD25^int^ mTreg Cell Produce More IL-10, IL-17A, and IFN-γ

The greater stability of the TSDR demethylation status and CD27 expression by naive Treg cells compared with CD25^int^ mTreg cell during expansion showed important differences between these populations. Critically, an enhanced stability of suppressive function after expansion in TAC by CD25^int^ mTreg cells compared with naive Treg cells prompted the measurement of the suppressive cytokine IL-10 and any divergence in its production between Treg cell populations.

**FIGURE 5:**
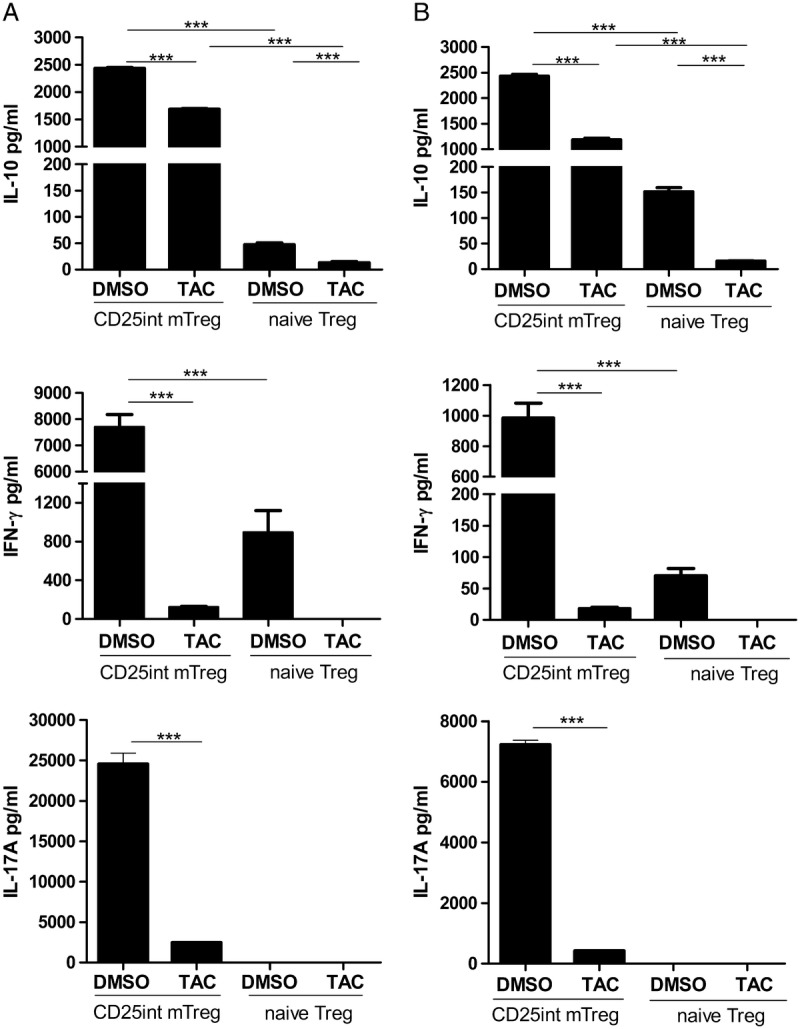
Cytokine production of Treg cell subpopulations. IL-10, IFN-γ, and IL-17A production by CD25^int^ mTreg and naive Treg cells after in vitro expansion in the presence of TAC or DMSO was determined in triplicate wells in 2 different donors (A and B). Bars represent means with SEM (***P < 0.001).

IL-10 production was accessed in supernatant after expansion of CD25^int^ mTreg and naive Treg cells in TAC or DMSO in 2 donors (Figure 5A, donor 1; B, donor 2). CD25^int^ mTreg produced substantially more IL-10 than naive Treg cells. Expansion when exposed to TAC significantly reduced IL-10 production by both Treg cell populations, with IL-10 production by naive Treg cell almost completely ablated but production by CD25^int^ mTreg remaining relatively high. Remaining IL-10 production when exposed to TAC by CD25^int^ mTreg cells may explain the greater stability of suppressive function of CD25^int^ mTreg cells compared with naive Treg cells.

Given the reduced percentage of CD25^int^ mTreg that possess a demethylated TSDR after expansion compared with naive Treg cells (Figure 3A), it is possible that CD25^int^ mTreg may have the potential to revert to an effector function. To test whether the reduced TSDR demethylation corresponds with enhanced effector function, we examined IFN-γ and IL-17A production after expansion. Indeed, CD25^int^ mTreg produce substantially greater levels of both IFN-γ and IL-17A compared with naive Treg (Figure 5). Tacrolimus significantly reduced both IL-17A and IFN-γ production (Figure 5).

## DISCUSSION

We show here that the CD4^+^CD25^+^CD127^−/lo^ population contains subpopulations of Treg with differential maintenance of TSDR demethylation status, CD27 expression, cytokine production, and ability to suppress when exposed to TAC during in vitro expansion.

Both CD25^int^ mTreg and naive Treg cell subpopulations had a similar high percentage of cells with a demethylated TSDR and FOXP3 expression before expansion. Loss of TSDR demethylation during expansion was restricted to CD25^int^ mTreg cells. It is possible that the CD25^int^ mTreg cell population is contaminated with memory Teff cell that expand faster than CD25^int^ mTreg cell to become the predominant constituent of cultured CD127^−/lo^CD25^int^CD45RA^−^ sorted cells after 14 days of polyclonal stimulation. Regulatory T cell TSDR demethylation status is described to be highly stable when cells are subjected to extended periods of in vitro expansion.^12^ It is therefore less likely that CD127^−/lo^CD25^+^CD45RA^−^ cells that are TSDR demethylated on day 0 undergo epigenetic alteration to possess a methylated TSDR during expansion. Both CD25^int^ mTreg and naive Treg cells express high levels of FOXP3 after expansion, yet the former may be accounted for by CD25^int^ Teff cell contaminants expressing FOXP3 after activation. The combined observations of a greater level of IL-10 production, a lack of a TSDR demethylated status, and expression of FOXP3 by CD25^int^ mTreg expanded cells strongly indicates that a large proportion of this population is comprised of de novo produced iTreg cells that may derive from Teff cells that contaminate the sorted population of day 0 and expand to form the main population constituent. Contaminating CD4^+^ Teff cells may have been converted to iTreg cells in TGF-β–rich culture conditions,^20^ derived from conventional Treg cells^21^ sorted alongside. The contaminating non-Treg population may have comprised a small constituent of the sorted CD25^int^ mTreg cell population on day 0 because the proportion of CD25^int^ mTreg and naive Treg with demethylated TSDR is similar at day 0. The dominant method by which naive Treg and CD25^int^ mTreg exert suppression may differ, given the differential level of IL-10 production and susceptibility of interference of suppression by TAC.

The large drop in the proportion of CD25^int^ mTreg with a demethylated TSDR post expansion, regardless of the exposure to TAC, identifies a considerable risk to adoptively transferring these cells into patients to tolerize allograft recipients. Although the CD25^int^ mTreg population showed superior IL-10 production and stability to suppress when expanded in the presence of TAC, the lack of a demethylated TSDR is known to identify cells with an unstable Treg phenotype that are susceptible to loosing suppressive function.^22^ Indeed, we observed that CD25^int^ mTreg produce substantially greater amounts of IL-17A and IFN-γ compared with naive Treg. Regulatory T cells with an unstable regulatory phenotype have been shown to have the potential to develop into pathogenic cells in mice^23^ and similar could be true for human Treg cells. It is possible that CD25^int^ mTreg cells, possibly containing a large proportion of cells derived from Teff cell contaminants on day 0 that expand faster than conventional Treg, will lose suppressive function after adoptive transfer to patients and damage the allograft.

We show that a naive Treg population may be sorted using surface markers and expanded to comprise a promising option for future studies to adoptively transfer Treg cells to tolerize an allograft. Despite some loss of Treg function in the presence of TAC, this population of cells nevertheless retains a high degree of suppressive function and importantly retains a TSDR demethylated status after expansion. This study indicates that sorting a population of naive Treg cells according to a CD4^+^CD45RA^+^CD25^int^CD127^−/lo^ will improve therapy beyond sorting the whole CD4^+^CD25^+^CD127^lo^ total Treg cell population. Future work may identify if sorting a mixture of naive Treg and CD25^hi^ mTreg will further enhance the production of a suitable Treg population for cellular therapy. Analysis of suppressive function in a freshly isolated nonexpanded total Treg cell population in patients undergoing haemodialysis showed a reduced suppressive function compared with healthy controls.^24^ Future studies may establish if the naive Treg population that we examined here has reduced capacity to suppress compared with the same population obtained from healthy subjects.

## Supplementary Material

SUPPLEMENTARY MATERIAL
