# Supplementary material for: CD45RA Distinguishes CD4+CD25+CD127−/low TSDR Demethylated Regulatory T Cell Subpopulations With Differential Stability and Susceptibility to Tacrolimus-Mediated Inhibition of Suppression
Source: Transplantation. 2016 Jul 28;101(2):302–9. doi: 10.1097/TP.0000000000001278 (PMC5265687; doi:10.1097/TP.0000000000001278)
Supplement: SUPPLEMENTARY MATERIAL [file tp-101-302-s001.pptx]

## Slide 1
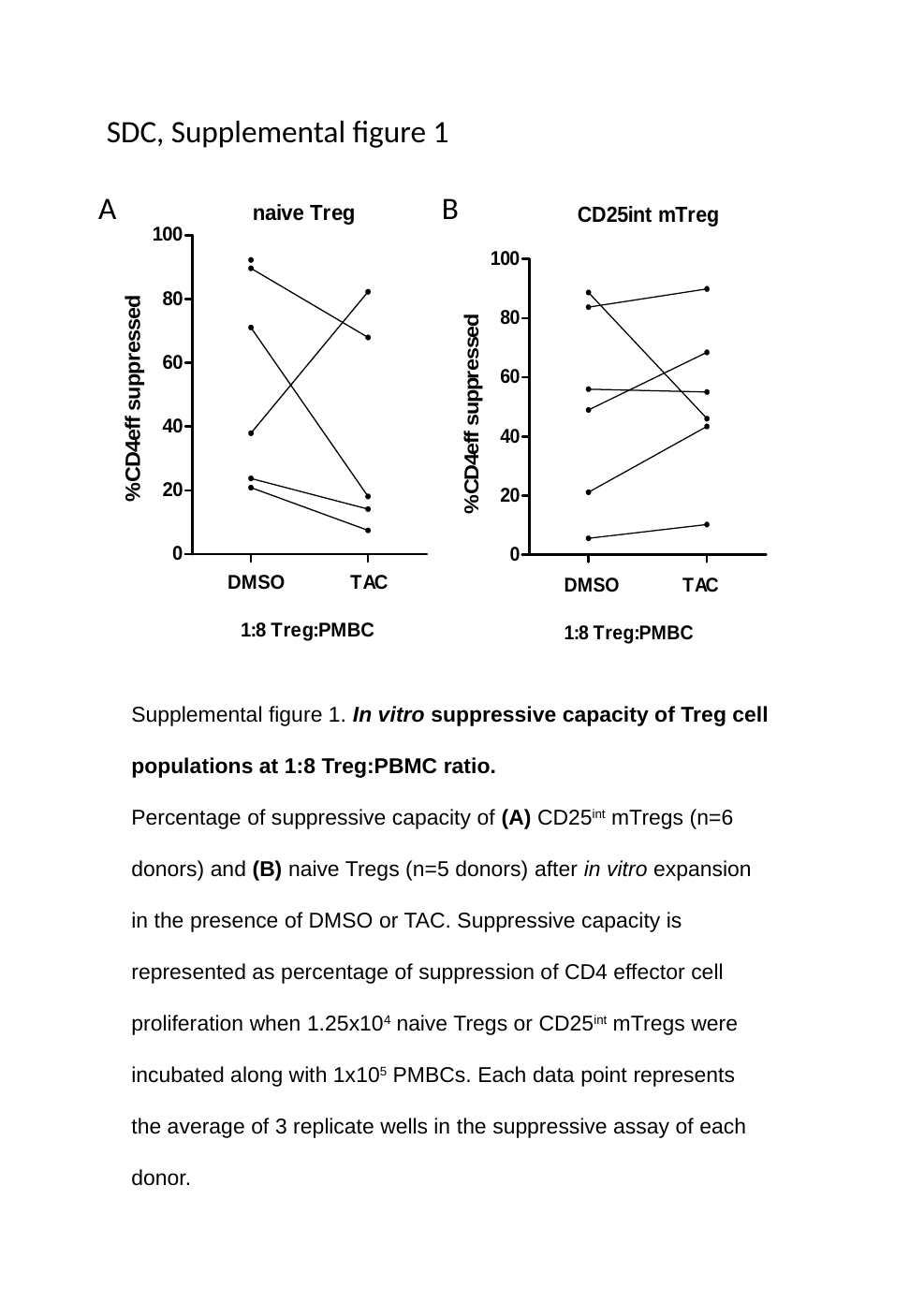

SDC, Supplemental figure 1
A
B
Supplemental figure 1. In vitro suppressive capacity of Treg cell populations at 1:8 Treg:PBMC ratio.
Percentage of suppressive capacity of (A) CD25int mTregs (n=6 donors) and (B) naive Tregs (n=5 donors) after in vitro expansion in the presence of DMSO or TAC. Suppressive capacity is represented as percentage of suppression of CD4 effector cell proliferation when 1.25x10^4^ naive Tregs or CD25int mTregs were incubated along with 1x10^5^ PMBCs. Each data point represents the average of 3 replicate wells in the suppressive assay of each donor.
